# Impact of Precapillary Component of Pulmonary Pressure in Tricuspid Transcatheter Edge-to-Edge Repair: Results From TRI-SPA

**DOI:** 10.1016/j.jacadv.2025.102153

**Published:** 2025-09-12

**Authors:** Antonio Sisinni, Xavier Freixa, Dabit Arzamendi, Vanessa Moñivas Palomero, Fernando Carrasco-Chinchilla, Manuel Pan, Luis Nombela-Franco, Isaac Pascual, Tomás Benito-González, Rodrigo Estévez-Loureiro

**Affiliations:** a Department of Cardiology, University Hospital Alvaro Cunqueiro, Vigo, Spain; b Cardiovascular Research Group, Department of Cardiology, University Hospital Alvaro Cunqueiro, Galicia Sur Health Research Institute (IIS Galicia Sur), Servizo Galego de Saude, University of Vigo, Vigo, Spain; c Department of Cardiology, IRCCS Policlinico San Donato, San Donato Milanese, Milan, Italy; d Department of Cardiology, Cardiovascular Institute, Hospital Clínic, Barcelona, Spain; e Division of Interventional Cardiology, Hospital de la Santa Creu i Sant Pau, Universitat Autónoma de Barcelona, Barcelona, Spain; f Department of Cardiology, University Hospital Puerta de Hierro-Majadahonda, Madrid, Spain; g Unidad de Gestión Clínica del Corazón, Hospital Universitario Virgen de la Victoria, CIBERCV, Instituto de Investigación Biomédica de Málaga (IBIMA), Universidad de Málaga (UMA), Málaga, Spain; h Department of Cardiology, Hospital Reina Sofía, Universidad de Córdoba, Instituto Maimónides de Investigación Biomédica de Córdoba (IMIBIC), Córdoba, Spain; i Cardiovascular Institute, Hospital Clínico San Carlos, IdISSC, Madrid, Spain; j Heart Area, Hospital Universitario Central de Asturias, Oviedo, Spain; k Department of Cardiology, University Hospital of León, León, Spain

**Keywords:** precapillary pulmonary hypertension, pulmonary hypertension, right heart catheterization, tricuspid regurgitation, tricuspid transcatheter edge-to-edge repair

**Figure:**
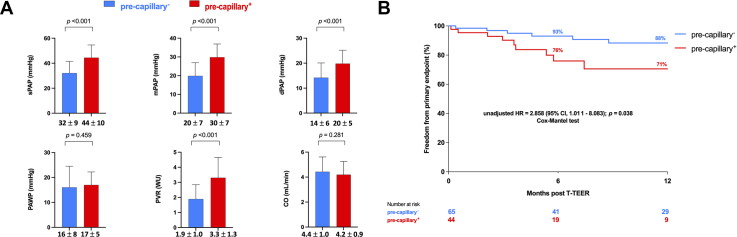

Severe tricuspid regurgitation (TR) is a relatively common valvular heart disease linked to unfavorable prognosis.^1^ Historically, invasive hemodynamics played a pivotal role in the management of valvular heart diseases, before giving way to noninvasive imaging techniques. Tricuspid transcatheter edge-to-edge repair (T-TEER) demonstrated safety and effectiveness in diminishing regurgitation severity and improving quality of life.^2^ In this analysis, we aim to investigate the prognostic significance of preprocedural right heart catheterization–derived pulmonary hypertension (PH) phenotyping, in patients undergoing T-TEER with dedicated devices.

**What is the clinical question being addressed?**

Among patients undergoing tricuspid transcatheter edge-to-edge repair, how does preprocedural right heart catheterization-derived pulmonary hypertension phenotyping predict prognosis compared with other phenotypes or absence of pulmonary hypertension?

**What is the main finding?**

The presence of preprocedural precapillary component of pulmonary hypertension predicted adverse clinical outcome after tricuspid transcatheter edge-to-edge repair.

The transcatheter tricuspid valve repair in Spain registry is an ongoing, single-arm, multicenter, retrospective registry of patients submitted to T-TEER, in 15 Spanish hospitals. Before the procedure, patients underwent transthoracic and transesophageal echocardiography to assess tricuspid valve leaflet morphology, evaluate anatomical suitability for T-TEER, and quantify the severity of TR, which ranged from none to torrential. Complete preprocedural hemodynamic assessment was performed. Pulmonary artery wedge pressure (PAWP), systolic/diastolic/mean pulmonary artery pressure (PAP) were measured. Cardiac output (CO) was obtained by either the indirect Fick method or thermodilution technique, depending on the specific study site protocol. Pulmonary vascular resistance (PVR) was determined as the difference between mean PAP and PAWP, divided by CO.

Patients were retrospectively divided into 2 groups according to the presence (precapillary^+^) or absence (precapillary^−^) of a precapillary component of PH, defined as PVR >2 WU.^3^ TR reduction by at least 1 grade defined procedural success. The primary endpoint was a composite of all-cause mortality, rehospitalization for heart failure, and tricuspid reintervention, at 1-year follow-up.

Event-free survival up to 1-year was evaluated according to the unadjusted Kaplan-Meier method and survival among subgroups was compared using the Cox–Mantel test. Univariable Cox proportional hazards regression was used to determine significant predictors of primary endpoint and of residual at least moderate TR. All statistical tests were 2-sided, and *P* values <0.05 were considered statistically significant. Statistical analyses were performed using SPSS software (version 28.0.0; SPSS Inc). Patients included in the study signed a written informed consent. Given the retrospective nature of the analysis; separate Internal Review Board approval was not required as per our local institutional policy. The investigation conforms to the principles outlined in the Declaration of Helsinki.

A total of 134 patients (mean age 72 ± 10 years, 69% female) were included. The precapillary^−^ group consisted of 79 (59%) individuals. Either symptomatic status or comorbidity burden were similar among study subgroups. Specifically, the proportion of patients in NYHA functional class III to IV was nearly identical between precapillary^+^ and precapillary^−^ groups (64% vs 63%; *P* = 0.999). Similarly, comorbidities such as atrial fibrillation (95% vs 86%; *P* = 0.154), chronic kidney disease (33% vs 29%; *P* = 0.705), and chronic obstructive pulmonary disease (22% vs 17%; *P* = 0.501) did not differ significantly. At least massive TR was present in 49% of participants overall, with a secondary etiology accounting for 79% of cases. Baseline echocardiographic parameters—including left ventricular ejection fraction (59% vs 58%; *P* = 0.299), tricuspid annular plane systolic excursion (24 mm vs 28 mm; *P* = 0.095), coaptation gap (5.7 mm in both groups; *P* = 0.866), and TR effective regurgitant orifice area (0.69 vs 0.65 cm^2^, *P* = 0.369)—were also comparable between precapillary^+^ and precapillary^−^ groups. Precapillary^+^ patients exhibited significantly higher preprocedural values of systolic/diastolic/mean PAP and, consistently with a priori definition, increased PVR. In contract, baseline PAWP and CO were similar between study groups (Figure 1A). Procedural success was achieved in 128 patients (96%), without difference among groups. Procedural complications and in-hospital mortality rates as well as predischarge residual TR degree were similar between study subgroups.

**Figure 1:**
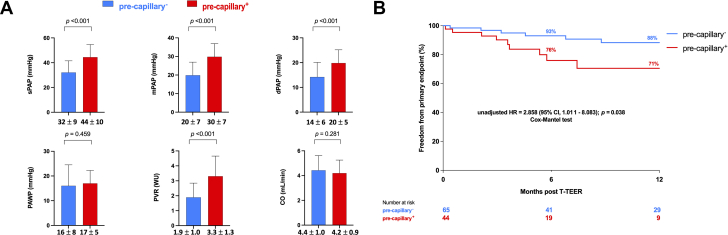
Invasive Hemodynamic Characteristics and Clinical Outcomes Among Study Groups (A) Baseline invasive hemodynamic profile in study groups. (B) One-year freedom from primary endpoint in study subgroups. CO = cardiac output; dPAP = diastolic PAP; mPAP = mean PAP; PAP = pulmonary artery pressure; PAWP = pulmonary artery wedge pressure; PVR = pulmonary vascular resistance; sPAP = systolic PAP; T-TEER = tricuspid transcatheter edge-to-edge repair.

At 1-year clinical and transthoracic echocardiographic follow-up significant improvement in NYHA functional class and TR severity was observed in the entire study cohort (NYHA functional class III/IV, decreased from 66% to 13%; *P* < 0.001; at least severe TR, decreased from 100% to 26%; *P* < 0.001). Residual severe or greater TR was present in 40% of precapillary^+^ patients, but in only 19% of precapillary^−^ patients at 1-year follow-up.

In the univariable analysis, precapillary^+^ was associated with residual severe or greater TR (OR: 2.836; 95% CI: 1.089-7.389; *P* = 0.033). At 1 year, the primary endpoint occurred more frequently in precapillary^+^ patients compared to precapillary^−^ patients (88% vs 71%; log-rank *P* = 0.038) (Figure 1B). After adjusting for a new risk score for in-hospital mortality prediction after isolated tricuspid valve surgery (TRI-SCORE) and type 1 tricuspid valve anatomy, precapillary^+^ phenotype (HR: 3.614; 95% CI: 1.207-10.825; *P* = 0.022) as well as residual severe or greater TR (HR: 4.559; 95% CI: 1.545-13.457; *P* = 0.007) were as independently associated with the primary endpoint.

In conclusion, our results indicate a substantial reduction in TR severity, and an improvement in NYHA-defined symptom status following T-TEER. The presence of preprocedural precapillary component of PH was associated with adverse clinical outcome (composite of all-cause mortality, rehospitalization for heart failure and tricuspid reintervention) after the procedure. Similarly, in a cohort of 236 patients with secondary TR undergoing T-TEER, significant precapillary contribution to PH was associated with 4-fold increase of mortality.^4^ Our results are potentially related to a higher rate of residual significant TR observed in precapillary^+^ population. Conversely, a subanalysis from EuroTR registry revealed that stratification of patients by PVR failed to demonstrate prognostic significance regarding primary outcome.^5^ Notably, unlike our analysis, patients with precapillary PH in the EuroTR registry exhibited a significantly lower incidence of signs right heart failure, lower mPAP, and PAWP, as well as smaller left and right ventricular dimensions and reduced tenting height and area compared to those with postcapillary or mixed PH. Despite these differences, invasive hemodynamic profiling remains a valuable tool for identifying patients who may benefit most from T-TEER. Further research is needed to better understand the impact of precapillary component of PH and outcomes after T-TEER.

## Funding support and author disclosures

Dr Freixa is a consultant and proctor for Abbott Vascular and Edwards Lifesciences. Dr Arzamendi is a consultant and proctor for Abbott Vascular and Edwards Lifesciences. Dr Carrasco-Chinchilla is proctor for Abbott Vascular. Dr Nombela-Franco is a consultant and proctor for Abbott Vascular. Isaac Pascual is proctor for Abbott Vascular. Dr Estevez-Loureiro is a consultant and proctor for Abbott Vascular, Edwards Lifesciences, Boston Scientific and Venus Medtech. All other authors have reported that they have no relationships relevant to the contents of this paper to disclose.
